# Thermoregulatory and plasma neurobiomarker responses to heat tolerance assessment in exertional heat illness and matched controls

**DOI:** 10.1113/EP093598

**Published:** 2026-08-24

**Authors:** Tom W. Palin, Jo Corbett, Lisa J. Hill, David R. Woods, Alex A. M. Gould, Barney Wainwright, James A. Roberts, Chloe N. Thomas, Daniel Snape, Carol House, Omar Tayari, Richard J. Elsworthy, James L. Mitchell, Andrew J. Roberts, John P. O'Hara, Michael J. Stacey

**Affiliations:** ^1^ Carnegie School of Sport Leeds Beckett University Leeds UK; ^2^ Extreme Environments Laboratory, School of Psychology, Sport, and Health Sciences, Faculty of Science and Health University of Portsmouth Portsmouth UK; ^3^ Department of Biomedical Sciences, School of Infection, Inflammation and Immunology, College of Medicine and Health University of Birmingham Birmingham UK; ^4^ Academic Department of Military Medicine Defence Medical Services Research and Innovation Birmingham UK; ^5^ Survival and Thermal Medicine Department Institute of Naval Medicine Gosport UK; ^6^ School of Sport, Exercise and Rehabilitation Sciences, College of Life and Environmental Sciences University of Birmingham Birmingham UK; ^7^ Army Recruit Health and Performance Research, Medical Branch, HQ Army Recruiting and Initial Training Command Ministry of Defence Upavon UK; ^8^ Department of Surgery and Cancer Imperial College London London UK

**Keywords:** blood–brain barrier, brain‐derived tau, heat stroke, traumatic brain injury

## Abstract

Exercise in the heat and elevated tissue temperature are associated with neuronal stress and changes in blood–brain barrier (BBB) function. More severe thermal insult may manifest as exertional heat illness (EHI) and complication by central nervous system (CNS) dysfunction may persist post‐EHI. We investigated neurobiomarkers associated with neuronal injury (brain‐derived tau, BD‐tau; neurofilament light, NfL; ubiquitin carboxyl‐terminal hydrolase isozyme L1, UCH‐L1) and BBB function (glial fibrillary acid protein, GFAP) in the peripheral blood of 34 recent (∼4 months) EHI patients and 30 Control individuals without prior EHI history, matched for variables influencing thermoregulation. Participants completed an exercise heat tolerance assessment (HTA), with neurobiomarkers measured pre‐ and post‐HTA. An equal number of participants (*n* = 6) in both EHI and Control groups showed thermoregulatory responses consistent with thermal intolerance. Pooled changes in BD‐tau, GFAP and UCH‐L1 showed moderate to large effect sizes (Cohen's *d* = 1.2, −1.3 and 0.75, respectively), including novel elevation in BD‐tau (median [interquartile range] 4.84 [4.10, 5.51] vs. 6.89 [5.69, 7.85] pg mL^−1^), whereas impact of HTA on NfL was negligible (Cohen's *d* = 0.03). No differences were evident in neurobiomarker response between EHI and Controls (time × group interaction *P* = 0.244), and no consistent associations between body core temperature and neurobiomarker concentrations were demonstrated. In conclusion, neurobiomarker responses to HTA were similar between EHI cases and Controls, indicating an absence of ongoing CNS injury in this patient cohort. However, variability in neurobiomarkers with HTA queries the general applicability of these biochemical surrogates in the management of brain insult associated with prior exercise and thermal stress.

## Abstract (highlights)

- **What is the central question of this study?**
  How do neurobiomarker surrogates of central nervous system (CNS) stress, measured in plasma, vary with exercise in the heat, and do values in patients affected previously by exertional heat illness (EHI) differ from those observed in matched Controls?
- **What is the main finding and its importance?**
  Heat tolerance assessment conducted >12 weeks post‐EHI associated with effect sizes that were large for brain‐derived tau and glial fibrillary acid protein, moderate for ubiquitin carboxyl‐terminal hydrolase isozyme L1, and negligible for neurofilament light. No variation in neurobiomarkers between EHI and Controls suggested equivalent CNS responses to exercise in the heat, reassuring against residual neurological insult in complication of prior EHI.

## 1 INTRODUCTION

Heat stress is a perennial feature of military service: arising under macroenvironmental challenge, with the microenvironmental stress of encapsulating dress states and personal protective equipment, and from physical exertion, including load carriage. Moderate heat stress impacts central nervous system (CNS) function adversely (Doohan et al., 2023; Gaoua et al., 2011); progressive hyperthermia associates with vasomotor paralysis, cerebral metabolic uncoupling and loss of cerebral pressure‐flow autoregulation (Cremer & Kalkman, 2007). With supervening incapacitation from heat stress (‘heat illness’), a spectrum of brain impairment is observed, ranging from mild, transient dysfunction to heat stroke, a life‐threatening state of hyperthermic encephalopathy. The term exertional heat illness (EHI) may be applied where heat illness arises during or soon after physical exertion, and exertional heat stroke represents its most severe manifestation. (Epstein & Yanovich, 2019).

Damage to the brain is evident in >90% of fatal military heat stroke cases (Haymaker et al., 1947). While lasting clinical sequelae, including a syndrome of chronic CNS dysfunction, have been reported in survivors, the underlying pathophysiology has been under‐investigated and is incompletely understood (Epstein, 1990; Wilkins et al., 2022; Yoneda et al., 2024). Other non‐communicable diseases that feature an acute neurological syndrome complicated by chronic cognitive impairment include multiple sclerosis (Zierfuss et al., 2024), autoimmune encephalitis (Homeyer et al., 2025) and traumatic brain injury (TBI), the latter a medical disorder of significant health and economic importance in the military‐age population (Simon et al., 2017; van Vliet et al., 2020). Shared mechanisms underlying the chronic manifestations of these diseases are thought to include neuroinflammation, degeneration and blood–brain barrier (BBB) disruption.

These various pathophysiological processes are reflected in molecular markers (so‐called ‘neurobiomarkers’) used increasingly for diagnosis and prognostication following brain insult. In the acute aftermath of TBI, the appearance of neurobiomarkers with CNS‐selectivity in peripheral blood shows distinct temporal trends (Zetterberg & Blennow, 2016) and, in longer term follow up, biochemical evidence for prior insult can be evidenced in patients sampled months and even years after their acute episode (Graham et al., 2025). A compromised BBB has been presented as a potential therapeutic target, not only to prevent or mitigate primary contemporaneous TBI effects, but also to alleviate chronic secondary CNS impairment (Simon et al., 2017; van Vliet et al., 2020). Relative effacement of BBB integrity has also been demonstrated with hyperthermia in animal models (Shohami et al., 1994; Yenari & Han, 2012), with higher local tissue temperatures leading to increased leakage of larger molecules from the CNS into the peripheral circulation, and vice versa (Kiyatkin & Sharma, 2009). These observations raise questions of whether increased permeability from thermal stress and insult would allow a greater volume and range of neurobiomarkers to be detectable in the peripheral circulation, and whether their longitudinal measurement post‐EHI might reveal perturbations comparable to those reported for TBI.

Quantification of serum S100 calcium‐binding protein B (S100B) – a neurotrophic factor sequestered in the CNS and released by astrocytes in the BBB – is currently advocated for the assessment of TBI and associated BBB disruption (Huibregtse et al., 2021; Wang et al., 2021). In health, serum concentrations have been shown to relate linearly with serum osmolality, both at rest (Marchi et al., 2003) and following exercise in the heat (Watson et al., 2005, 2006). An additional, though less consistent, association between S100B and core body temperature (*T*_c_) measured by rectal thermistor has also been reported (Watson et al., 2005, 2006). However, extra‐neuronal reservoirs of S100B are known to exist in pulmonary, cardiac, connective and adipose tissues, and concern has been expressed for lack of CNS specificity during strenuous physical exertion (Koh & Lee, 2014). This may explain why, in our previous work, assay of serum S100B did not discriminate between collapsed marathon participants diagnosed with EHI and other runners who completed the same event successfully (Stacey et al., 2022).

Neurobiomarkers characterised and brought to commercial market since the emergence of S100B include glial fibrillary acid protein (GFAP) – which, like S100B, is a marker of astrocyte (astroglial) activation and BBB function – and surrogates of neuronal insult such as ubiquitin carboxyl‐terminal hydrolase isozyme L1 (UCH‐L1), tau proteins and neurofilament light (NfL) (Huibregtse et al., 2021; Wang et al., 2021). Assay of S100B, GFAP and UCH‐L1 are now recommended as part of standard clinical care for people with acute TBI. Launched in 2025, the international initiative for blood biomarker profiling in TBI stratification also recognises the association of a large proportion of TBI cases (up to 20%) with athletic or recreational activities, and directs further investigation into the impact of physical exertion on biomarkers, particularly when these are known to be expressed by non‐CNS tissues (Manley et al., 2025).

Blood levels of GFAP, UCH‐L1 and NfL show differing responses to acute bouts of strenuous physical exertion, according to the timing of sampling, the molecule assayed and the intervention selected (Bazarian et al., 2023; Joisten et al., 2021; Uddin et al., 2025). As for S100B, plasma concentrations of tau are known to be influenced by exercise (Kawata et al., 2018; Neselius et al., 2013), such that total tau lacks sufficient specificity for CNS tissues to serve as a reliable surrogate of brain insult in this context. More recently an assay has been developed to quantify brain‐derived tau (BD‐tau), which incorporates only tau isoforms specific to the CNS (Gonzalez‐Ortiz, Dulewicz et al., 2023, Gonzalez‐Ortiz, Turton et al., 2023). This presents the opportunity to re‐examine relationships between neuronal stress, BBB function and exertional hyperthermia described previously (Watson et al., 2005, 2006), but potentially without the confounding influence of extra neuronal release associated with S100B (Koh & Lee, 2014).

Thus, the primary aim of the present work was to assess how exercise‐heat stress would influence the appearance of BD‐tau, GFAP, NfL and UCH‐L1 in peripheral blood, including an exploration of blood neurobiomarker concentrations and their associations with *T*_c_ response. The model we adopted incorporated historic military EHI cases and healthy service person controls who undertook a military occupational Heat Tolerance Assessment (HTA), as described in allied reporting (Gould et al., 2025). As the potential for persistence of neurobiomarker elevation in the weeks following EHI has not been studied, this approach allowed us to address the subsidiary hypotheses that, versus matched controls, EHI cases would evidence relatively elevated neurobiomarker responses at rested baseline and with the stimulation of HTA. Based on the temporal trends in these molecules described post‐TBI (Zetterberg & Blennow, 2016), we specifically anticipated that EHI cases with underlying CNS dysfunction would show increased GFAP and NfL at rested baseline, and exaggerated BD‐tau and UCH‐L1 responses shortly after HTA completion.

## 2 METHODS

### 2.1 Ethical approval

Ethical approval was granted by the United Kingdom (UK) Ministry of Defence Research Ethics Committee (2093/MODREC/21). The study was conducted in accordance with the *Declaration of Helsinki*, and all participants gave written informed consent. Military volunteers were recruited from patients who had been incapacitated while exercising under heat stress (EHI) and referred to the UK military Heat Illness Clinic (HIC) between February 2022 and March 2023, and from among personnel serving at an Army Unit local to the HIC, who were aged between 18 and 45 years old and had no previous history of EHI (Controls).

### 2.2 Participants

Patients were approached for recruitment if they had been either (a) hospitalised for EHI with end‐organ insult, defined as CNS disturbance – including seizure, Glasgow Coma Scale (GCS) < 8 for 15 min or longer – and/or biochemical evidence of other organ/tissue damage (graded, per HIC policy, as Moderate severity EHI, or Severe if critical care had been required in hospital), or (b) incapacitated by more than one episode of EHI (Recurrent EHI). Exclusion criteria applied to the referral episode were hyponatraemia, exertional rhabdomyolysis and lack of documented hyperthermia. For both EHI and Controls, pre‐existing cardiovascular, metabolic and respiratory medical conditions precluded participation. However, individuals known to have consumed potential modifiers of gastrointestinal integrity (e.g., prebiotics, probiotics and/or antibiotics) within the previous 3 months, or non‐steroidal anti‐inflammatory medications (NSAIDs) and/or stool altering medications (e.g., laxatives and anti‐diarrhoea), within 1 month of the experimental protocol – who were excluded from the previously reported study of non‐neurobiomarker data (Gould et al., 2025) – were retained in our analysis, as these agents were not anticipated to impact upon the CNS processes under investigation.

Each cohort was matched for factors known to influence thermoregulatory responses and underwent identical procedures, as described previously (Gould et al., 2025; House et al., 2023; Stacey et al., 2025). Anthropometry, estimation of maximal oxygen uptake, and HTA were conducted sequentially. All exercise was conducted in a climatic chamber with conditions configured to target a dry bulb temperature of 34°C and 44% relative humidity.

### 2.3 Exercise testing

Peak ${\dot{V}}_{O_{2}}$ was determined as an incremental running test to volitional exhaustion, with participants wearing shorts, T‐shirt and trainers. HTA consisted of treadmill marching at 60% intensity of ${\dot{V}}_{O_{2}\mathrm{peak}}$, initially with load carriage and additional clothing worn. Subsequent phases of the HTA were initiated with progressive removal of load and clothing as follows:

Phase 1 (0–30 min): Volunteers carried a 14–16 kg rucksack and walked on the treadmill with the carried load, speed, and gradient set to elicit a work intensity equivalent to 60% ${\dot{V}}_{O_{2}\mathrm{peak}}$. After 5 min, expired gas was analysed to ensure the correct work intensity was achieved, and the speed and gradient were adjusted if necessary. The speed and gradient then remained the same for the rest of the test.

Phase 2 (30–45 min): At 30 min, the rucksack and jacket were removed.

Phase 3 (45–90 min): At 45 min the T‐shirt was removed. Volunteers continued to walk until 60 min and were then stopped if a plateau (i.e., two consecutive readings the same) or fall in rectal temperature occurred; if rectal temperature was still rising, the volunteer continued until a plateau occurred or 90 min had elapsed.

Participants were withdrawn from HTA no sooner than 60 min, but before *T*_c_ (measured using rectal thermistor) exceeded 39.5°C. The HTA was extended for up to a further 30 min (total duration 90 min) to allow participants to demonstrate a plateau in both *T*_c_ and heart rate. Achievement of this dual plateau and completion of at least 60 min of the protocol was defined as thermal tolerance, as previously reported for this HTA (Gould et al., 2025; House et al., 2023; Stacey et al., 2025).

### 2.4 Sampling

Additional sampling included 1.5 mL of urine collected during baseline anthropometry (osmolality measured by freezing point depression, Gonotec, Berlin, Germany). A resting venous blood sample taken from an antecubital vein into lavender‐topped vacutainers containing EDTA (Becton Dickinson, Franklyn Lakes, NJ, USA), before (Pre‐HTA) and within 15 min following HTA (post‐HTA). Measurement of haemoglobin and haematocrit, in triplicate (Hawksley & Sons Ltd, Lancing, UK), allowed for the calculation of Δplasma volume (Dill & Costill, 1974). Blood was centrifuged (1500 *g*, 10 min) and frozen to −80°C for transportation. Plasma samples were thawed at room temperature, and samples were analysed in duplicate for GFAP, UCH‐L1, NfL and BD‐Tau using the Simoa® Neurology 4‐Plex D Advantage PLUS kit on a Quanterix SR‐X machine (Quanterix Corp., Bilerica, MA, USA) according to the manufacturer's instructions. Briefly, this was achieved by mixing and loading the samples onto the Quanterix SR‐X machine for bead‐based digital immunoassay processing. This followed the process of incubating the samples with capture beads, detecting antibody binding, enzyme labelling, and measuring the levels of single molecules. High and low controls were included on each assay. All samples were within the limit of detection (LOD) and limits of quantification (LLOQ) defined by the manufacturer.

### 2.5 Statistical analysis

Data analyses were conducted using statistical software (Prism version 8.1.0, GraphPad Software, San Diego, CA, USA). Pairwise comparisons of raw data were made for volunteer characteristics and to characterise the HTA exposure (Table 1), using Student's *t*‐test or a non‐parametric equivalent. A power calculation was not attempted, as the sample size was determined by the previous Gould et al. (2025) study. Effect sizes consequent upon HTA exposure (pooled EHI and Controls, Pre‐HTA versus post‐HTA) were calculated for each neurobiomarker and were reported as Cohen's *d* (*d* < 0.2: trivial; >0.2: small; >0.5: moderate; >0.8: large). Neurobiomarkers were assessed for normality using the Shapiro–Wilk test, and those found to be non‐parametrically distributed were logarithmically transformed for two‐way ANOVA (EHI status × Time). Univariate linear correlation analysis was performed separately in Patients and Controls, again using log‐transformed neurobiomarker data, using Pearson's or Spearman's correlation coefficient for parametric and non‐parametric data, respectively. Results are presented as means ± SD, or median [interquartile range] for non‐parametric data. *P *< 0.05 was adopted for significance, adjusted for multiple comparisons (Šidák's test) for *post hoc* comparison (EHI versus Controls) in two‐way ANOVA.

**TABLE 1:** Physiological and performance characteristics of EHI and controls.

Variable	EHI (*n* = 34)	Controls (*n* = 30)	*P*
**Physiological characteristics**			
Age (years)	28 ± 6	27 ± 5	0.784
Height (cm)	180.2 ± 7	181.5 ± 7.8	0.477
Body mass (kg)	87.4 ± 8.8	86.4 ± 10.3	0.699
Baseline BMI (kg m^−2^)	26.9 ± 2.8	26.2 ± 2.6	0.286
Body fat percentage (%)	15.3 ± 4.5	15.4 ± 3.6	0.955
Body surface area (m^2^)	2.1 ± 0.1	2.1 ± 0.2	0.960
${\dot{V}}_{O_{2}\max}$ (mLkg^−1^ min^−1^)	51.4 ± 7	52.4 ± 5.7	0.547
Urine osmolality (mOsmkg^−1^)	511 ± 330	667 ± 352	0.073
**HTA measures**			
Duration (min)	63 ± 9	62 ± 7	0.603
Phase 1 exercise intensity (% ${\dot{V}}_{O_{2}\max}$)	59.4 ± 1.8	58.7 ± 2	0.149
Phase 2 exercise intensity (% ${\dot{V}}_{O_{2}\max}$)	52.6 ± 3.5	51.8 ± 2.9	0.346
Sweat rate (L h^−1^)	1.3 ± 0.4	1.4 ± 0.3	0.864
End exercise *T*_c_ (°C)	38.9 ± 0.4	38.8 ± 0.3	0.137
End exercise *T*_skin_ (°C)	33.2 ± 3.3	33.9 ± 1.1	0.309
End exercise HR (bpm)	165 ± 17	156 ± 17	0.043

Abbreviations: BMI, body mass index; HR, heart rate; HTA, heat tolerance assessment; *T*_c_, core body temperature; *T*_skin_, skin temperature; ${\dot{V}}_{O_{2}\max}$, maximum rate of oxygen consumption.

## 3 RESULTS

Conditions observed in the climatic chamber were ambient temperature 34.7 ± 0.7°C, humidity 44.4 ± 3.1%. Table 1 characterises EHI (*n* = 34 males; 80% affected by end‐organ insult, as categorised from referral information; 29% having had more than one lifetime episode of heat illness; median time from EHI to HTA 112 days, range 83–238 days) and Controls (*n* = 30 males), including standard data captured from clinical application of the HTA. An equal number in each group (*n* = 6) failed to demonstrate thermal tolerance per clinic guidelines, that is, attainment of a plateau in both *T*_c_ and heart rate within the time allowed.

Following adjustment for changes in plasma volume with HTA, large effect sizes on neurobiomarker concentrations (EHI and Controls combined) were observed for BD‐tau, which increased from pre‐HTA to post‐HTA (4.84 [4.10, 5.51] vs. 6.89 [5.69, 7.85] pg mL^−1^, Cohen's *d* = 1.2), and for GFAP, which decreased (32.16 [23.62, 46.36] vs. 24.78 [20.65, 33.76] pg mL^−1^, Cohen's *d* = −1.3). Effect size was only trivial for NfL (2.95 [2.38, 4.01] vs. 2.87 [2.27, 3.70] pg mL^−1^, Cohen's *d* = 0.03), whereas a moderate effect was evident for UCH‐L1 (12.91 [8.49, 20.01] vs. 21.41 [14.89, 27.99] pg mL^−1^, Cohen's *d* = 0.75).

**FIGURE 1:**
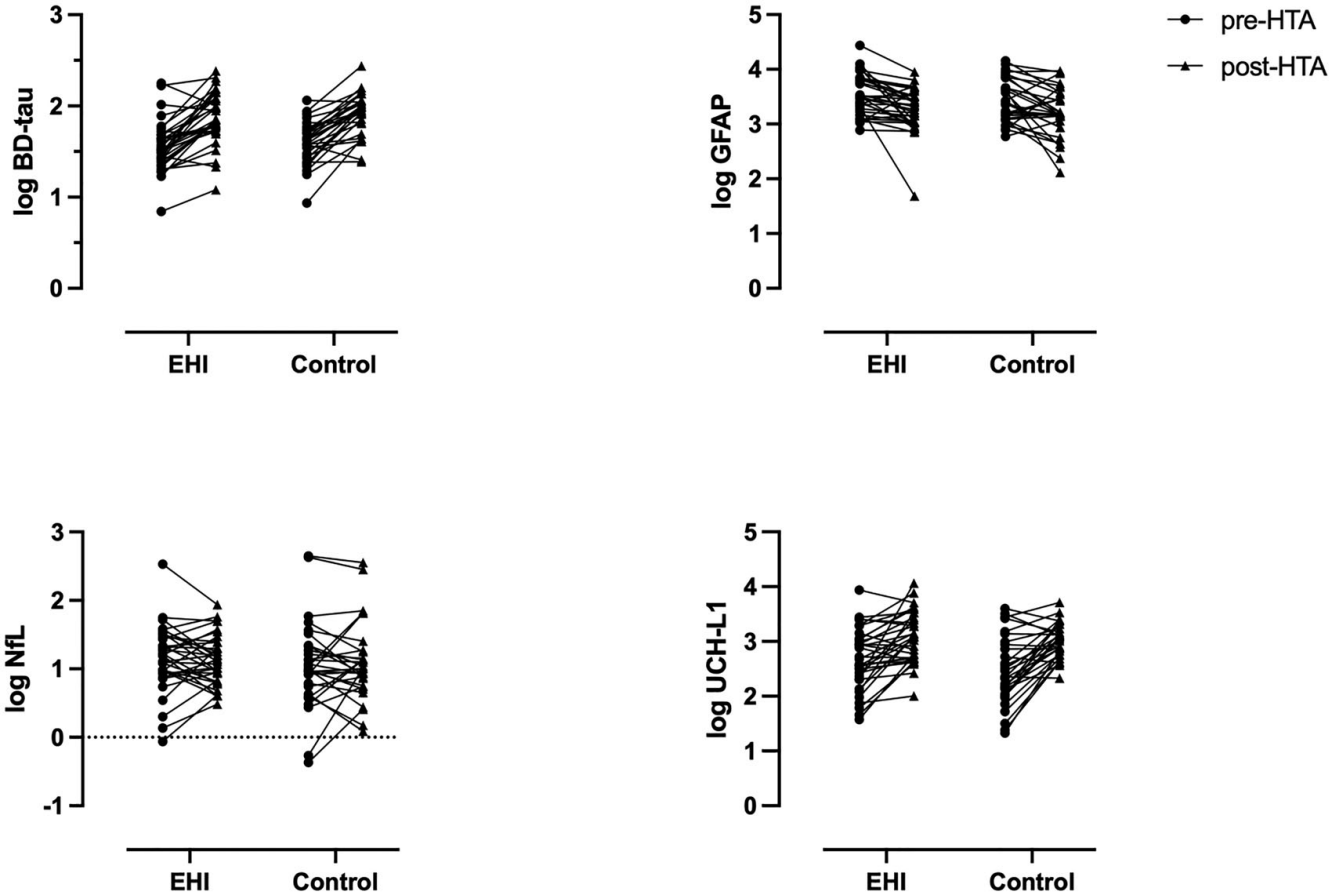
Log‐transformed neurobiomarker concentrations, adjusted for changes in plasma volume, from venous blood sampling pre‐HTA and post‐HTA heat tolerance assessment, in EHI and controls. BD‐tau, brain derived Tau; GFAP, glial fibrillary acid protein; NfL, neurofilament light; UCH‐L1, ubiquitin carboxyl‐terminal hydrolase isozyme L1.

We assessed the levels of neurobiomarkers (NfL, BD‐Tau, UCH‐L1 and GFAP) in participants who had an EHI compared to Controls and found no effect of group (EHI vs. Controls, *P* = 0.204, 2‐way ANOVA of log‐transformed values) and no interaction between time and group (*P* = 0.244) was evident for any neurobiomarker (Figure 1). As anticipated from calculation of effect sizes, main effects of time (pre‐HTA to post‐HTA) were evident for BD‐tau, GFAP and UCH‐L1, with significant rise in BD‐tau (*F* = 112.8, *P *< 0.0001) and UCH‐L1 (*F* = 53.92, *P *< 0.0001) and decrease in GFAP (*F* = 22.42, *P *< 0.0001). No main effect of group and no interaction was shown for NfL (*P* = 0.504).

The results of correlation analyses for neurobiomarkers vs. *T*_c_ (at rest and post‐HTA) are displayed in Table 2, indicating only three significant linear associations, which were modest in strength and inconsistent in their associations (*r* = −0.48 for NfL, versus resting *T*_c_ only; *r* = 0.43 for both BD‐tau and GFAP, versus *T*_c_ post‐HTA only).

**TABLE 2:** Pearson correlation matrix of coefficients relating core body temperature (*T*_c_) with log‐transformed neurobiomarker concentrations, in exertional heat illness (EHI) cases and controls, sampled at rest (pre‐HTA) and immediately following (post‐HTA) heat tolerance assessment.

	EHI (*n* = 34)		Controls (*n* = 30)	
	pre‐HTA	post‐HTA	pre‐HTA	post‐HTA
**BD‐tau**	*r* = −0.24, *P* = 0.172	*r* = 0.14, *P* = 0.423	*r* = 0.35, *P* = 0.055	***r* = 0.43,** ***P* = 0.019**
**GFAP**	*r* = 0.12, *P* = 0.193	*r* = −0.17, *P* = 0.346	*r* = −0.24, *P* = 0.193	***r* = 0.43,** ***P* = 0.019**
**NfL**	*r* = −0.26, *P* = 0.136	*r* = −0.28, *P* = 0.112	***r* =** −**0.48,** ***P* = 0.008**	*r* = −0.24, *P* = 0.207
**UCH‐L1**	*r* = −0.15, *P* = 0.408	*r* = 0.23, *P* = 0.196	*r* = −0.09, *P* = 0.650	*r* = 0.09, *P* = 0.627

Values shown in bold are considered statistically significant (*P* < 0.05).

Abbreviations: BD‐tau, brain derived Tau; GFAP, glial fibrillary acid protein; NfL, neurofilament light; ns, non‐significant association (*P* > 0.05); UCH‐L1, ubiquitin carboxyl‐terminal hydrolase isozyme L1.

## 4 DISCUSSION

This work is, to our knowledge, the first published investigation of neurobiomarkers in EHI convalescence, and provides the first comparison of dynamic neurobiomarker responses in EHI cases and matched controls. Our results include differential variation concentrations of the four molecules assessed in relation to hyperthermia, including the first reporting of the CNS‐specific molecule BD‐tau with exercise of any kind. However, our findings of no difference in plasma concentrations for EHI cases versus Controls, either at rested baseline or immediately following HTA, appear to reject our hypothesis of a relative neurobiomarker increase following EHI.

### 4.1 Variation in neurobiomarkers with exercise in the heat

In military cases of heat stroke, characteristic histopathological changes in non‐CNS tissues and organ systems are approximated by biochemical measurement of peripheral blood surrogates such as creatinine and liver transaminases (Ward et al., 2020). Clinicians rely upon temporal trends in the excursion of these markers to evidence the diagnosis of EHI, assist in prognostication and inform decisions on return‐to‐play or ‐duties in affected athletes and uniformed service personnel (DeGroot et al., 2022), including the use of HTA described in the present work. Thus, the concept of peripheral blood assay of brain‐specific peptides, providing a window into CNS function in EHI, holds considerable appeal for this population and other occupational and recreational groups (Schlader et al., 2022).

Assay of BD‐tau – which showed a large effect of HTA in the present work – has the potential to more accurately reflect increased passage of neuronal markers from the CNS into peripheral blood, versus neurobiomarkers such as S100B, which lacks specificity for CNS tissues, or neuron specific enolase, which is subject to significant artefactual confounding from haemolysis (Babkina et al., 2024). The first reported assay of BD‐tau was accomplished only recently (Gonzalez‐Ortiz, Turton et al., 2023), with the molecule found to show high correlation between cerebrospinal fluid (CSF) and plasma concentrations. Non‐CNS tau derived in the periphery originates in cardiac, liver and kidney tissues and is highly abundant, comprising 80% of total plasma tau (Barthélemy et al., 2020); these extra‐neuronal sources have hampered the interpretation of previous work showing excursions in total‐tau with exercise alone, that is, in the absence of acute TBI or other intercurrent pathology (Kawata et al., 2018). However, the BD‐tau assay targets a large peptide‐insert in peripheral tau – resulting from the transcription of an extra exon (exon 4a) of the microtubule‐associated protein tau (MAPT) gene – thereby unmasking the underlying brain‐derived signal. Thus, the elevation that we report reflects this CNS‐specific component, most likely passing from the brain to the periphery under the influence of exercise and associated thermal stress.

Other findings in the present work are congruent with a decrease in GFAP (Bazarian et al., 2023) and an increase for UCH‐L1 (Bazarian et al., 2023; Uddin et al., 2025) observed by other investigators, who have recently addressed the question of release – in the absence of overt brain injury – in relation to medium duration aerobic exercise and strength/conditioning work (45 to 120 min). These findings, and those of the present work, differ from our own previous observations, which indicated no significant changes in GFAP and UCH‐L1 with successful marathon performance (median finishing time 4 h) (Stacey et al., 2023).

Conflicting results may reflect differences in the nature of the exercising exposures selected, as well as the timing of sample collection and nature of the assay selected. For example, GFAP is thought to decline acutely under the influence of cortisol (Bazarian et al., 2023), and the HTA protocol described in the present work is known to elicit a rise in adrenocortical hormones, including cortisol (Stacey et al., 2025). Transient depression in GFAP levels may have been captured by sampling within 15 min of HTA completion, whereas the equivalent window in our previous work, allowing up to 30 min post‐marathon for sampling, may have been too wide to capture this change (and also too early for the peak in GFAP known to arise from around 20 h following brain insult in TBI studies) (Huibregtse et al., 2021; Wang et al., 2021). Furthermore, by using the highly sensitive Quanterix Simoa® assay, which employs single molecule counting technology, we may have detected changes in UCH‐L1 post‐HTA that were not evident with the less sensitive enzyme linked immunosorbent assay (ELISA) used to analyse samples from our study of the marathon. As UCH‐L1 has been reported to show a progressive rise in the hours following exercise (Bazarian et al., 2023; Uddin et al., 2025), sampling later than 15 or 30 min may have yielded values that were different from marathon baseline in our previous work, or higher again than the elevated values observed post‐HTA in the present study.

### 4.2 Neurobiomarkers in EHI and Controls

Of the neurobiomarkers assayed in our selected panel, GFAP (Graham et al., 2025) and NfL (Shahim et al., 2020) are known to show resting elevation in the peripheral blood of patients many months after TBI. However, in the present work, we saw no difference between EHI and Control, either before or after HTA. The high specificity of NfL for neuronal axons, which are perhaps at greater risk of injury from the shearing forces involved in TBI than the hyperthermic encephalopathy of heat stroke, suggests its association with a molecular signature for EHI would be less likely. However, any differential change in BBB integrity between EHI cases and Controls could conceivably result in higher expression of GFAP, an astroglial marker, and greater egress of NfL from the CNS into peripheral blood, and therefore our findings argue against residual brain pathology of this nature complicating EHI in the cases observed. This provides a degree of reassurance in the face of rodent data indicating convalescent elevation in brain chemokines and cytokines following heat stroke (Biedenkapp & Leon, 2013), which have been used to support a hypothesis that post‐EHI inflammation may sensitise the CNS to future stressors, perhaps conferring heat intolerance in survivors.

### 4.3 Neurobiomarkers and *T*_c_ response

Consistent relationships between neurobiomarkers and *T*_c_ were also absent in the results of our study, despite historic observations indicating a role for exertional hyperthermia in degrading the integrity of the BBB in healthy participants (Watson et al., 2005, 2006). Where present, the correlations we observed were only weak to moderate in strength, were limited to Controls only and were not consistently present pre/post‐HTA. While this might point to altered mechanisms of neurobiomarker transportation from the CNS with heat stress or following EHI, the finding of no differences in absolute plasma concentrations for any of the four molecules in post‐HTA sampling renders this a hypothesis to address in future work, perhaps with serial sampling extended into the hours following exercise‐heat stress, in order to more fully capture temporal differences in extra‐CNS release. As none of our participants was assessed sooner than 12 weeks following EHI, and none were sufficiently unwell at the time of their illness to have required critical care in a hospital environment, we believe this hypothesis would be best tested in a cohort affected by more severe EHI in aggregate, and at an earlier stage in their recovery, in order to have the best chance of re‐capitulating any relevant pathology.

### 4.4 Limitations

In relation to the specific issue raised above, and our results in general, it should be observed that the HTA protocol described is geared towards thermal compensability, and the achievement of a plateau in thermoregulatory parameters before thermo‐effector mechanisms reach their expected asymptote, that is, at around *T*_c_ of 39.5°C. This may not have afforded sufficient stimulus for the anticipated changes in BBB to manifest as differences in neurobiomarkers entering peripheral blood for sampling. For example, in the rodent model of selective brain heating cited above, GFAP positivity in CNS structures was not evident until local tissue temperature gradually increased above approximately 38.5°C, and intracerebral oedema in keeping with significant permeability did not begin to appear until 39.0°C (Kiyatkin & Sharma, 2009). These values are not directly comparable with our reporting, given the use of human participants, the tendency of brain temperature to run approximately 0.5°C higher than *T*_c_ and the isolated thermal stimulus in the animal data. Nevertheless, we believe that a more thermally provoking protocol may have been required to more completely address our primary aim of establishing neurobiomarker variation in this context, and our secondary aim of exploring any differential neurobiomarker response in EHI.

Other limitations of the present work include lack of serial sampling post‐HTA, with our protocol only affording opportunity to collect blood soon after the completion of HTA. Previous work following exercise performed in health, and in patients acutely affected by TBI, highlights different temporal trends in the peripheral blood appearance of each molecule (Kawata et al., 2016). These observations have been taken to indicate alternative mechanisms of transfer from the CNS than allowed for in previous speculation on the significance of changes in blood concentrations with exercise. The more recent discovery of the glymphatic pathway (Iliff et al., 2012) casts further doubt on the ability of a single molecule to purely reflect BBB when measured in this context. In any event, we acknowledge that sampling at just this one relatively early time point post‐HTA may have meant that potential changes over time or between groups were not identified in our study. Furthermore, as serum osmolality was not measured, these data do not allow us to judge the accuracy of historical conclusions regarding the dependence of BBB integrity on changes in tonicity with exercise (Watson et al., 2005, 2006).

Lastly, as a reflection of the equal pass rate for the HTA observed, neurobiomarker responses did not differ between EHI cases and Controls. Neither outcome was anticipated but may reflect the substantial time allowed for recovery before assessment in the EHI group (112 days, range 83–238 days) and absence from the cohort of severe EHI cases requiring critical care admission. This difference, plus the non‐significant trend to higher baseline urine osmolality in Controls – which might favour relative BBB effacement – could account for equivalent HTA outcome and failure of a discriminant neurobiomarker finding. Therefore, future work should more carefully match EHI patients and controls for baseline hydration and extracellular tonicity.

### 4.5 Conclusions

We have demonstrated variation in the acute peripheral blood response to exertional‐heat stress, consistent with differential transfer of neurobiomarkers from the CNS. Further evidence is provided to query the general applicability of neurobiomarkers in the management of brain insult, without first considering the specific impact of prior exercise and thermal stress. In EHI characterised by end‐organ injury and/or recurrent disease, lack of variation versus Controls supports the premise that substantial CNS insult is not perpetuated beyond 12 weeks post‐injury. Nevertheless, future work should incorporate extended, serial sampling post‐exercise, in order to allow for the possibility of a discriminant neurobiomarker signal emerging over time.

## AUTHOR CONTRIBUTIONS

Tom W. Palin, Jo Corbett, David R. Woods, Alex A. M. Gould, Barney Wainwright, Daniel Snape, Carol House, Omar Tayari, Andrew J. Roberts, John P. O'Hara and Michael J. Stacey conceived and designed research. Alex A. M. Gould, Carol House and Omar Tayari performed experiments at the INM. Tom W. Palin, Lisa J. Hill, James A. Roberts, Chloe N. Thomas and Richard J. Elsworthy performed sample analysis. Tom W. Palin, Lisa J. Hill, David R. Woods, Barney Wainwright, James A. Roberts, Chloe N. Thomas, Daniel Snape, John P. O'Hara and Michael J. Stacey analysed data. Tom W. Palin, Jo Corbett, David R. Woods, Barney Wainwright, Daniel Snape, John P. O'Hara and Michael J. Stacey interpreted the data. Tom W. Palin and Michael J. Stacey prepared figures. Tom W. Palin and Michael J. Stacey drafted the manuscript. Tom W. Palin, Jo Corbett, Lisa J. Hill, David R. Woods, Barney Wainwright, James A. Roberts, Chloe N. Thomas, Daniel Snape, Andrew J. Roberts, John P. O'Hara and Michael J. Stacey edited and revised the manuscript. All authors have read and approved the final version of this manuscript and agree to be accountable for all aspects of the work in ensuring that questions related to the accuracy or integrity of any part of the work are appropriately investigated and resolved. All persons designated as authors qualify for authorship, and all those who qualify for authorship are listed.

## CONFLICT OF INTEREST

No competing interests declared.

## GENERATIVE AI STATEMENT

The authors confirm that no artificial intelligence tools, including large language models (LLMs), were used in the drafting or revision of this manuscript. All content was conceived, written, and approved solely by the authors.

## Data Availability

Further information supporting the findings reported in this paper was previously made available in the paper by Gould et al. (2025). Additional data supporting the findings of this study are available from the corresponding author upon reasonable request and subsequent to the approval of the UK Ministry of Defence.

## References

[eph70395-bib-0001] Babkina, A. S. , Lyubomudrov, M. A. , Golubev, M. A. , Pisarev, M. V. , & Golubev, A. M. (2024). Neuron‐specific enolase—what are we measuring? International Journal of Molecular Sciences, 25(9), 5040. 38732258 10.3390/ijms25095040 PMC11084499

[eph70395-bib-0002] Barthélemy, N. R. , Horie, K. , Sato, C. , & Bateman, R. J. (2020). Blood plasma phosphorylated‐tau isoforms track CNS change in Alzheimer's disease. Journal of Experimental Medicine, 217(11), e20200861. 32725127 10.1084/jem.20200861 PMC7596823

[eph70395-bib-0003] Bazarian, J. J. , Abar, B. , Merchant‐Borna, K. , Pham, D. L. , Rozen, E. , Mannix, R. , Kawata, K. , Chou, Y. , Stephen, S. , & Gill, J. M. (2023). Effects of physical exertion on early changes in blood‐based brain biomarkers: Implications for the acute point of care diagnosis of concussion. Journal of Neurotrauma, 40(7–8), 693–705. 36200628 10.1089/neu.2022.0267 PMC10061333

[eph70395-bib-0004] Biedenkapp, J. C. , & Leon, L. R. (2013). Increased cytokine and chemokine gene expression in the CNS of mice during heat stroke recovery. American Journal of Physiology‐Regulatory, Integrative and Comparative Physiology, 305(9), R978–R986. 24026076 10.1152/ajpregu.00011.2013

[eph70395-bib-0005] Cremer, O. L. , & Kalkman, C. J. (2007). Cerebral pathophysiology and clinical neurology of hyperthermia in humans. Progress in Brain Research, 162, 153–169. 17645919 10.1016/S0079-6123(06)62009-8

[eph70395-bib-0006] DeGroot, D. W. , O'Connor, F. G. , & Roberts, W. O. (2022). Exertional heat stroke: An evidence based approach to clinical assessment and management. Experimental Physiology, 107(10), 1172–1183. 35771080 10.1113/EP090488

[eph70395-bib-0007] Dill, D. B. , & Costill, D. L. (1974). Calculation of percentage changes in volumes of blood, plasma, and red cells in dehydration. Journal of Applied Physiology, 37(2), 247–248. 4850854 10.1152/jappl.1974.37.2.247

[eph70395-bib-0008] Doohan, M. A. , Watzek, J. T. , King, N. , White, M. J. , & Stewart, I. B. (2023). Does increased core temperature alter cognitive performance during exercise‐induced heat strain? A narrative review. Journal of Applied Physiology, 135(1), 35–52. 37141422 10.1152/japplphysiol.00070.2023

[eph70395-bib-0009] Epstein, Y. (1990). Heat intolerance: Predisposing factor or residual injury? Medicine & Science in Sports & Exercise, 22(1), 29–35. 2406544

[eph70395-bib-0010] Epstein, Y. , & Yanovich, R. (2019). Heatstroke. New England Journal of Medicine, 380(25), 2449–2459. 31216400 10.1056/NEJMra1810762

[eph70395-bib-0011] Gaoua, N. , Grantham, J. , El Massioui, F. , Girard, O. , & Racinais, S. (2011). Cognitive decrements do not follow neuromuscular alterations during passive heat exposure. International Journal of Hyperthermia, 27(1), 10–19. 21070138 10.3109/02656736.2010.519371

[eph70395-bib-0012] Gonzalez‐Ortiz, F. , Dulewicz, M. , Ashton, N. J. , Kac, P. R. , Zetterberg, H. , Andersson, E. , Yakoub, Y. , Hanrieder, J. , Turton, M. , Harrison, P. , Nellgård, B. , Karikari, T. K. , & Blennow, K. (2023). Association of serum brain‐derived tau with clinical outcome and longitudinal change in patients with severe traumatic brain injury. JAMA Network Open, 6(7), e2321554–e2321554. 37399012 10.1001/jamanetworkopen.2023.21554 PMC10318474

[eph70395-bib-0013] Gonzalez‐Ortiz, F. , Turton, M. , Kac, P. R. , Smirnov, D. , Premi, E. , Ghidoni, R. , Benussi, L. , Cantoni, V. , Saraceno, C. , Rivolta, J. , Ashton, N. J. , Borroni, B. , Galasko, D. , Harrison, P. , Zetterberg, H. , Blennow, K. , & Karikari, T. K. (2023). Brain‐derived tau: A novel blood‐based biomarker for Alzheimer's disease‐type neurodegeneration. Brain, 146(3), 1152–1165. 36572122 10.1093/brain/awac407 PMC9976981

[eph70395-bib-0014] Gould, A. A. , Walsh, N. P. , Tipton, M. J. , Zurawlew, M. J. , Tayari, O. , House, C. , Delves, S. K. , Robson, S. C. , Shute, J. J. , & Watts, J. E. (2025). Faecal microbiome, gastrointestinal integrity, inflammation and thermoregulation in recent exertional heat illness patients and matched controls. Experimental Physiology, 111(2), 403–425. 40551374 10.1113/EP092849 PMC12857521

[eph70395-bib-0015] Graham, N. S. , Blissitt, G. , Zimmerman, K. , Orton, L. , Friedland, D. , Coady, E. , Laban, R. , Veleva, E. , Heslegrave, A. J. , Zetterberg, H. , Schofield, S. , Fear, N. T. , Boos, C. J. , Bull, A. M. J. , Bennett, A. , & Sharp, D. J. (2025). Poor long‐term outcomes and abnormal neurodegeneration biomarkers after military traumatic brain injury: The ADVANCE study. Journal of Neurology, Neurosurgery & Psychiatry, 96(2), 105–113. 39393903 10.1136/jnnp-2024-333777 PMC11877046

[eph70395-bib-0016] Haymaker, W. , Malamud, N. , & Custer, R. (1947). Heat stroke; a clinic‐pathologic study of 125 fatal cases. Journal of Neuropathology and Experimental Neurology, 6, 209–211. 20340260

[eph70395-bib-0017] Homeyer, M. A. , Falck, A. , Li, L. Y. , & Prüss, H. (2025). From immunobiology to intervention: Pathophysiology of autoimmune encephalitis. Seminars in Immunology, 78, 101955. 40267699 10.1016/j.smim.2025.101955

[eph70395-bib-0018] House, C. , Stacey, M. , Woods, D. , Allsopp, A. , & Roiz De Sa, D. (2023). Procedure for assessing patients referred to the UK's military heat illness clinic: A case series. BMJ Military Health, 169(4), 310–315. 34266969 10.1136/bmjmilitary-2021-001875

[eph70395-bib-0019] Huibregtse, M. E. , Bazarian, J. J. , Shultz, S. R. , & Kawata, K. (2021). The biological significance and clinical utility of emerging blood biomarkers for traumatic brain injury. Neuroscience & Biobehavioral Reviews, 130, 433–447. 34474049 10.1016/j.neubiorev.2021.08.029 PMC8511294

[eph70395-bib-0020] Iliff, J. J. , Wang, M. , Liao, Y. , Plogg, B. A. , Peng, W. , Gundersen, G. A. , Benveniste, H. , Vates, G. E. , Deane, R. , Goldman, S. A. , Nagelhus, E. A. , & Nedergaard, M. (2012). A paravascular pathway facilitates CSF flow through the brain parenchyma and the clearance of interstitial solutes, including amyloid β. Science Translational Medicine, 4(147), 147ra111–147ra111. 10.1126/scitranslmed.3003748 PMC3551275 22896675

[eph70395-bib-0021] Joisten, N. , Rademacher, A. , Warnke, C. , Proschinger, S. , Schenk, A. , Walzik, D. , Knoop, A. , Thevis, M. , Steffen, F. , & Bittner, S. (2021). Exercise diminishes plasma neurofilament light chain and reroutes the kynurenine pathway in multiple sclerosis. Neurology: Neuroimmunology & Neuroinflammation, 8, e982. 33782190 10.1212/NXI.0000000000000982 PMC8054957

[eph70395-bib-0022] Kawata, K. , Liu, C. Y. , Merkel, S. F. , Ramirez, S. H. , Tierney, R. T. , & Langford, D. (2016). Blood biomarkers for brain injury: What are we measuring? Neuroscience & Biobehavioral Reviews, 68, 460–473. 27181909 10.1016/j.neubiorev.2016.05.009 PMC5003664

[eph70395-bib-0023] Kawata, K. , Rubin, L. H. , Wesley, L. , Lee, J. H. , Sim, T. , Takahagi, M. , Bellamy, A. , Tierney, R. , & Langford, D. (2018). Acute changes in plasma total tau levels are independent of subconcussive head impacts in college football players. Journal of Neurotrauma, 35(2), 260–266. 29073820 10.1089/neu.2017.5376

[eph70395-bib-0024] Kiyatkin, E. A. , & Sharma, H. S. (2009). Permeability of the blood–brain barrier depends on brain temperature. Neuroscience, 161(3), 926–939. 19362131 10.1016/j.neuroscience.2009.04.004 PMC2694729

[eph70395-bib-0025] Koh, S. X. , & Lee, J. K. (2014). S100B as a marker for brain damage and blood–brain barrier disruption following exercise. Sports Medicine, 44(3), 369–385. 24194479 10.1007/s40279-013-0119-9

[eph70395-bib-0026] Manley, G. T. , Dams‐O'Connor, K. , Alosco, M. L. , Awwad, H. O. , Bazarian, J. J. , Bragge, P. , Corrigan, J. D. , Doperalski, A. , Ferguson, A. R. , Mac Donald, C. L. , Menon, D. K. , Mcnett, M. M. , Van Der Naalt, J. , Nelson, L. D. , Pisică, D. , Silverberg, N. D. , Umoh, N. , Wilson, L. , Yuh, E. L. , … Yurgelun‐Todd, D. (2025). A new characterisation of acute traumatic brain injury: The NIH‐NINDS TBI classification and nomenclature initiative. The Lancet Neurology, 24(6), 512–523. 40409315 10.1016/S1474-4422(25)00154-1

[eph70395-bib-0027] Marchi, N. , Rasmussen, P. , Kapural, M. , Fazio, V. , Kight, K. , Mayberg, M. R. , Kanner, A. , Ayumar, B. , Albensi, B. , Cavaglia, M. , & Janigro, D. (2003). Peripheral markers of brain damage and blood‐brain barrier dysfunction. Restorative Neurology and Neuroscience, 21(3–4), 109–121. 14530574 PMC4066375

[eph70395-bib-0028] Neselius, S. , Zetterberg, H. , Blennow, K. , Randall, J. , Wilson, D. , Marcusson, J. , & Brisby, H. (2013). Olympic boxing is associated with elevated levels of the neuronal protein tau in plasma. Brain Injury, 27(4), 425–433. 23473386 10.3109/02699052.2012.750752

[eph70395-bib-0029] Schlader, Z. J. , Davis, M. S. , & Bouchama, A. (2022). Biomarkers of heatstroke‐induced organ injury and repair. Experimental Physiology, 107(10), 1159–1171. 35654394 10.1113/EP090142 PMC9529995

[eph70395-bib-0030] Shahim, P. , Politis, A. , van der Merwe, A. , Moore, B. , Ekanayake, V. , Lippa, S. M. , Chou, Y. Y. , Pham, D. L. , Butman, J. A. , Diaz‐Arrastia, R. , Zetterberg, H. , Blennow, K. , Gill, J. M. , Brody, D. L. &. , & Chan, L. (2020). Time course and diagnostic utility of NfL, tau, GFAP, and UCH‐L1 in subacute and chronic TBI. Neurology, 95(6), e623–e636. 32641529 10.1212/WNL.0000000000009985 PMC7455355

[eph70395-bib-0031] Shohami, E. , Novikov, M. , & Horowitz, M. (1994). Long term exposure to heat protects against brain damage induced by closed head injury in the rat. Restorative Neurology and Neuroscience, 6(2), 107–112. 21551737 10.3233/RNN-1994-6204

[eph70395-bib-0032] Simon, D. W. , McGeachy, M. J. , Bayır, H. , Clark, R. S. , Loane, D. J. , & Kochanek, P. M. (2017). The far‐reaching scope of neuroinflammation after traumatic brain injury. Nature Reviews Neurology, 13(3), 171–191. 28186177 10.1038/nrneurol.2017.13 PMC5675525

[eph70395-bib-0033] Stacey, M. J. , Hill, N. E. , Parsons, I. T. , Wallace, J. , Taylor, N. , Grimaldi, R. , Shah, N. , Marshall, A. , House, C. , O'Hara, J. P. , Brett, S. J. , & Woods, D. R. (2022). Relative changes in brain and kidney biomarkers with Exertional Heat Illness during a cool weather marathon. PLOS ONE, 17(2), e0263873. 35176088 10.1371/journal.pone.0263873 PMC8853487

[eph70395-bib-0034] Stacey, M. J. , House, C. , de Sa, D. R. , Brett, S. J. , Boot, C. , Teggert, A. , Allsopp, A. J. , & Woods, D. R. (2025). Adrenal steroid hormone responses to exercise under thermal stress: Potential role for nonclassic congenital adrenal hyperplasia in heat illness susceptibility. Physiological Reports, 13(6), e70272. 40110968 10.14814/phy2.70272 PMC11923862

[eph70395-bib-0035] Stacey, M. J. , Leckie, T. , Fitzpatrick, D. , Hodgson, L. , Barden, A. , Jenkins, R. , Galloway, R. , Weller, C. , Grivas, G. V. , Pitsiladis, Y. , Richardson, A. J. , & Woods, D. R. (2023). Neurobiomarker and body temperature responses to recreational marathon running. Journal of Science and Medicine in Sport, 26(11), 566–573. 37777396 10.1016/j.jsams.2023.09.011

[eph70395-bib-0036] Uddin, N. , Scott, J. , Nixon, J. , Patterson, S. D. , Kidgell, D. , Pearce, A. J. , Waldron, M. , & Tallent, J. (2025). The effects of exercise, heat‐induced hypo‐hydration and rehydration on blood–brain‐barrier permeability, corticospinal and peripheral excitability. European Journal of Applied Physiology, 125(2), 535–550. 39340668 10.1007/s00421-024-05616-x PMC11829906

[eph70395-bib-0037] van Vliet, E. A. , Ndode‐Ekane, X. E. , Lehto, L. J. , Gorter, J. A. , Andrade, P. , Aronica, E. , Gröhn, O. , & Pitkänen, A. (2020). Long‐lasting blood‐brain barrier dysfunction and neuroinflammation after traumatic brain injury. Neurobiology of Disease, 145, 105080. 32919030 10.1016/j.nbd.2020.105080

[eph70395-bib-0038] Wang, K. K. , Munoz Pareja, J. C. , Mondello, S. , Diaz‐Arrastia, R. , Wellington, C. , Kenney, K. , Puccio, A. M. , Hutchison, J. , Mckinnon, N. , Okonkwo, D. O. , Yang, Z. , Kobeissy, F. , Tyndall, J. A. , Büki, A. , Czeiter, E. , Pareja Zabala, M. C. , Gandham, N. , & Berman, R. (2021). Blood‐based traumatic brain injury biomarkers–Clinical utilities and regulatory pathways in the United States, Europe and Canada. Expert Review of Molecular Diagnostics, 21(12), 1303–1321. 34783274 10.1080/14737159.2021.2005583

[eph70395-bib-0039] Ward, M. D. , King, M. A. , Gabrial, C. , Kenefick, R. W. , & Leon, L. R. (2020). Biochemical recovery from exertional heat stroke follows a 16‐day time course. PLOS ONE, 15(3), e0229616. 32130237 10.1371/journal.pone.0229616 PMC7055888

[eph70395-bib-0040] Watson, P. , Black, K. E. , Clark, S. C. , & Maughan, R. J. (2006). Exercise in the heat: Effect of fluid ingestion on blood‐brain barrier permeability. Medicine and Science in Sports and Exercise, 38(12), 2118–2124. 17146318 10.1249/01.mss.0000235356.31932.0a

[eph70395-bib-0041] Watson, P. , Shirreffs, S. M. , & Maughan, R. J. (2005). Blood‐brain barrier integrity may be threatened by exercise in a warm environment. American Journal of Physiology‐Regulatory, Integrative and Comparative Physiology, 288(6), R1689–R1694. 15650123 10.1152/ajpregu.00676.2004

[eph70395-bib-0042] Wilkins, D. , O'Sullivan, O. , Sayer, J. , Penny, L. , Roiz de Sa, D. , Ellis, H. , & Dharm‐Datta, S. (2022). Neurological rehabilitation following heat illness in the UK armed forces. BMJ Military Health, 168(4), 320–323. 33087541 10.1136/bmjmilitary-2020-001602

[eph70395-bib-0043] Yenari, M. A. , & Han, H. S. (2012). Neuroprotective mechanisms of hypothermia in brain ischaemia. Nature Reviews Neuroscience, 13(4), 267–278. 22353781 10.1038/nrn3174

[eph70395-bib-0044] Yoneda, K. , Hosomi, S. , Ito, H. , Togami, Y. , Oda, S. , Matsumoto, H. , Shimazaki, J. , Ogura, H. , & Oda, J. (2024). How can heatstroke damage the brain? A mini review. Frontiers in Neuroscience, 18, 1437216. 39450121 10.3389/fnins.2024.1437216 PMC11499184

[eph70395-bib-0045] Zetterberg, H. , & Blennow, K. (2016). Fluid biomarkers for mild traumatic brain injury and related conditions. Nature Reviews Neurology, 12(10), 563–574. 27632903 10.1038/nrneurol.2016.127

[eph70395-bib-0046] Zierfuss, B. , Larochelle, C. , & Prat, A. (2024). Blood–brain barrier dysfunction in multiple sclerosis: Causes, consequences, and potential effects of therapies. The Lancet Neurology, 23(1), 95–109. 38101906 10.1016/S1474-4422(23)00377-0

